# Metal-Phenolic Network-Coated Hyaluronic Acid Nanoparticles for pH-Responsive Drug Delivery

**DOI:** 10.3390/pharmaceutics11120636

**Published:** 2019-11-28

**Authors:** Jung Min Shin, Gwan Hyun Choi, Seok Ho Song, Hyewon Ko, Eun Sook Lee, Jae Ah Lee, Pil J. Yoo, Jae Hyung Park

**Affiliations:** 1 School of Chemical Engineering, College of Engineering, Sungkyunkwan University, Suwon 16419, Korea; jmabcd@skku.edu (J.M.S.); chh7894@naver.com (G.H.C.); thd328@skku.edu (S.H.S.); tizi9057@gmail.com (H.K.); jaeah_92@naver.com (J.A.L.); 2 Department of Health Science and Technology, SAIHST, Sungkyunkwan University, Suwon 16419, Korea; eunsook.lee0203@gmail.com; 3 SKKU Advanced Institute of Nanotechnology, SAINT, Sungkyunkwan University, Suwon 16419, Korea; 4 Biomedical Institute for Convergence at SKKU, Sungkyunkwan University, Suwon 16419, Korea

**Keywords:** metal-phenolic network, hyaluronic acid, pH-responsive particle, drug delivery

## Abstract

Although self-assembled nanoparticles (SNPs) have been used extensively for targeted drug delivery, their clinical applications have been limited since most of the drugs are released into the blood before they reach their target site. In this study, metal-phenolic network (MPN)-coated SNPs (MPN-SNPs), which consist of an amphiphilic hyaluronic acid derivative, were prepared to be a pH-responsive nanocarrier to facilitate drug release in tumor microenvironments (TME). Due to their amphiphilic nature, SNPs were capable of encapsulating doxorubicin (DOX), chosen as the model anticancer drug. Tannic acid and FeCl_3_ were added to the surface of the DOX-SNPs, which allowed them to be readily coated with MPNs as the diffusion barrier. The pH-sensitive MPN corona allowed for a rapid release of DOX and effective cellular SNP uptake in the mildly acidic condition (pH 6.5) mimicking TME, to which the hyaluronic acid was exposed to facilitate receptor-mediated endocytosis. The DOX-loaded MPN-SNPs exhibited a higher cytotoxicity for the cancer cells, suggesting their potential use as a drug carrier in targeted cancer therapy.

## 1. Introduction

Nanomedicine, an offshoot of nanotechnology, has been studied extensively for use in biomedical applications including targeted therapy and precise diagnosis of intractable diseases [1,2,3,4,5]. Several formulations are currently on the market or in clinical trials for cancer therapy, including Genexol^®^ PM (paclitaxel-encapsulated polymeric micelle) and SP1049C (doxorubicin-encapsulated polymeric micelle) [6,7,8].

These formulations exhibit superior therapeutic effects and reduced side effects, primarily ascribed to their unique properties, which improve the hydrophobic drug’s water solubility and/or enhance the drug’s accumulation at the tumor site by the enhanced permeation and retention effect [9]. However, these formulations have shown limited beneficial effects since the drugs are often released before they reach the tumor tissue, requiring the development of a drug delivery system with a reduced initial burst release of the anticancer drugs [10,11].

In recent years, stimuli-responsive polymers have emerged as drug carriers for cancer therapy since they can be designed to release the anticancer drugs in response to tumor-specific microenvironments (TMEs) such as a low pH, abnormal levels of reactive oxygen species, and hypoxic conditions [12,13,14,15]. These polymeric materials, however, have been chemically modified through multiple processes, which are often costly and time-consuming [16,17]. There are also concerns about the toxicity of fragments generated by cleaving stimuli-responsive linkers or moieties in nanomedicines [18,19].

Meanwhile, hyaluronic acid (HA), composed of N-acetyl glucosamine and D-glucuronic acid, is an anionic polysaccharide that is abundant in synovial fluid and the extracellular matrix in the biological system [20,21]. Owing to its specific binding affinity for CD44-overexpressing cancer cells, HA has been used extensively to prepare amphiphilic derivatives, capable of self-assembling into the nano-sized particles as a potential drug carrier for targeted cancer therapy [22,23,24]. However, these HA nanoparticles (HANPs), physically assembled in an aqueous condition, possess limited stability under physiological conditions and release a significant amount of the drug into the bloodstream.

**Figure 1:**
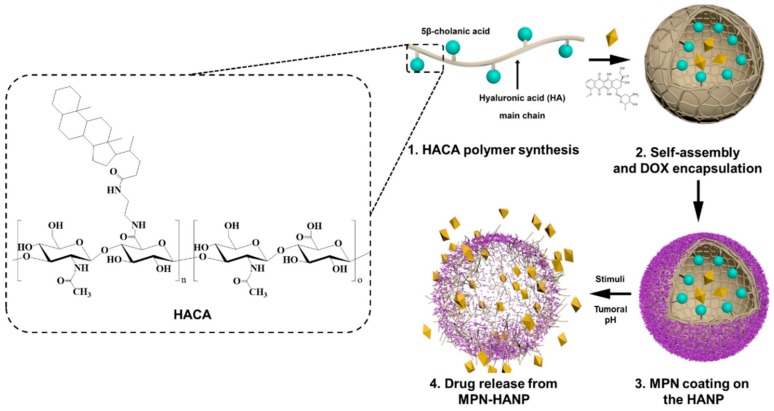
Schematic illustration of a metal-phenolic network-coated hyaluronic nanoparticle (MPN-HANP) designed for pH-sensitive drug release.

This study aimed to develop HA-based nanoparticles that are physiologically stable and will hold the drugs until they reach the TMEs. Using a simple and rapid means of fabrication method, we prepared metal-phenolic network (MPN)-coated HANPs (MPN-HANPs) as a pH-sensitive nanocarrier for a hydrophobic drug (Figure 1). MPN is a supramolecular coordination structure, consisting of polyphenol derivatives and metal ions [25]. Since MPN formation is driven by chelation, film assembly–disassembly behavior is strongly affected by pH in an aqueous solution. Owing to its unique features such as low toxicity and pH-dependent physicochemical properties, MPN has been extensively investigated as a drug carrier in combination with mesoporous nanoparticles and nanocomplexes [26,27,28,29,30]. Doxorubicin (DOX), chosen as the model anticancer drug, was encapsulated into the HANPs using an emulsion method. To generate pH-responsive MPN coats on the HANPs’ surface, tannic acid (TA) and Fe^3+^ ions were used as the organic and metal ion components, respectively. The physiochemical characteristics of the MPN-HANPs were evaluated and their in vitro release behavior of DOX was investigated at different pH conditions. The cellular uptake and the cytotoxicity of the DOX-loaded MPN-HANPs (DOX-MPN-HANPs) were also assessed in order to better understand their cancer therapy potential.

## 2. Materials and Methods

### 2.1. Materials

HA (MW = 2.34 × 10^5^ g/mol) was purchased from Lifecore Biomedical Inc. (Chaska, MN, USA). 5β-cholanic acid (CA), DOX·HCl, *N*-(3-Dimethylaminopropyl)-*N*′-ethylcarbodiimide hydrochloride (EDC), *N*-hydroxysuccinimide (NHS), formamide, dimethylformamide (DMF), TA, and iron (III) chloride hexahydrate (FeCl_3_·6H_2_O) were purchased from Sigma Aldrich Co. (St. Louis, MO, USA) and were used as received. RPMI medium and fetal bovine serum (FBS) were purchased from Capricorn Scientific GmbH (Ebsdorfergrund, Germany). Dulbecco’s Phosphate-Buffered Saline (DPBS) was purchased from WELGENE Inc. (Kyungsan, Korea) for in vitro experiments. The water used in this study was purified by using the Young Lin aquaMAX-ultra water purification system (Anyang, Korea).

### 2.2. Synthesis of Amphiphilic HA Derivative (HACA)

HACA was prepared using EDC/NHS chemistry. For this chemistry, the amine derivative of CA was prepared as previously reported [31,32,33]. Then, the aminated CA was chemically conjugated to the HA backbone. Briefly, HA (100 mg, 0.264 mmol) was dissolved in 25 mL of formaldehyde and stirred in a round bottom flask. EDC (40.4 mg, 0.211 mmol) and NHS (24.27 mg, 0.211 mmol), dissolved in formamide, were slowly added to the HA solution. The aminated CA (21.2 mg, 0.0527 mmol), dissolved in 25 mL of DMF, was added dropwise to the HA solution. The reaction was allowed to proceed for 24 h at room temperature. The solution was dialyzed against distilled water (DW) and methanol for two days using membrane tubing (molecular weight cut off [MWCO]: 12–14 kDa), followed by lyophilization. The freeze-dried HACA was stored at −20 °C before further application.

### 2.3. Fabrication of MPN-HANPs

To fabricate the HANP solution, the HACA was dispersed in distilled water. Then, 5 µL of TA aqueous solution (40 mg/mL) and 5 µL of FeCl_3_ aqueous solution (6.5 mg/mL) were slowly added to 490 µL of the HANP (0.4 mg/mL) solution, followed by gentle mixing to form an MPN layer on the HANPs. After the formation of the MPN layer on the nanoparticular surface, the residual TA and Fe^3+^ complexes were removed by ultrafiltration using Amicon Ultra Centrifugal Filter Units (Millipore, Burlington, MA, USA), according to manufacturer’s instructions.

### 2.4. Characterization of MPN-HANPs

The chemical structure of the HACA and the degree of substitution of the CA were assessed by using ^1^H-NMR (Varian Unity 500MHz spectrometer, Palo Alto, CA, USA), in which C*D*_3_O*D* and *D*_2_O were used as the solvent. The hydrodynamic size and zeta potential of the HANPs and MPN-HANPs were observed by dynamic light scattering (DLS) using a Zetasizer Nano ZS90 (Malvern Ins. Worcestershire, UK) with a He-Ne 633nm laser at a 90° detection angle. The morphologies of the HANPs and MPN-HANPs were characterized by using HR-TEM (JEOL-2100F, Tokyo, Japan) operated at an accelerating voltage of 200 kV. For TEM images, the samples were dispersed in deionized water (DIW) and dropped onto a 300-mesh copper grid. The nanoparticles were then treated with 1% uranyl acetate for negative staining. The UV-visible (UV-Vis) absorption spectra were measured on a UV-vis spectrophotometer (Optizen 3220UV, Mecasys Co., Ltd., Daejeon, Korea). To investigate the stability of the nanoparticles, the changes in the scattering intensity of the HANPs and MPN-HANPs were assessed in the presence of sodium dodecyl sulfate (SDS) as a typical micelle-destabilizing agent. Briefly, the HANP and MPN-HANPs (1 mg/mL in DIW) were incubated with or without SDS (2.5 mg/mL). The resulting solutions were kept at room temperature and the scattering intensity of each solution was recorded using DLS as a function of time.

### 2.5. Preparation of DOX-MPN-HANP

The DOX-HANPs were prepared using an emulsion (water-in-oil) method. DOX·HCl (2 mg) was dissolved in dichloromethane, and three equivalents of triethylamine were slowly added while mixing. The DOX solution was added into an aqueous HANP (18 mg) solution to generate a water-in-oil emulsion. The emulsion was stirred overnight to evaporate the organic solvent in dark conditions. The solution was filtered using a 0.80 µm syringe filter to remove unloaded DOX aggregations. After filtration, the solution was dialyzed against DIW using membrane tubing (MWCO = 1 kDa), followed by lyophilization. The lyophilized DOX-HANPs (1.2 mg) were dispersed in 1 mL of DIW and the DOX was dissolved by adding 2 mL of DMF. After filtration of solution, the DOX amount of each of the samples was determined using the DOX standard curve. The amount of DOX loaded in the DOX-HANPs was determined using a UV-Vis spectrophotometer (Optizen 3220UV, Mecasys Co., Ltd., Daejeon, Korea) at a wavelength of 480 nm. To fabricate the DOX-MPN-HANPs, the MPN was coated on the DOX-HANPs in an identical manner to the MPN-HANPs. The hydrodynamic size and zeta potential of the DOX-MPN-HANPs were evaluated using a Zetasizer Nano ZS90, as described earlier.

### 2.6. pH-Sensitive Release Behavior of DOX-MPN-HANP

To assess pH-sensitive drug release from the MPN-HANPs, the DOX-MPN-HANPs were dispersed in two different phosphate buffers (pH 7.4 and 6.5) and transferred to dialysis membrane tubes (MWCO = 3.5 kDa). The membranes were placed in conical tubes prefilled with 30 mL of phosphate buffer, one at pH 7.4 and the other at pH 6.5, and incubated at 100 rpm in a shaking water bath at 37 °C. At predetermined time points, each membrane was transferred to a new conical tube prefilled with the phosphate buffer, and the amount of released DOX was measured using a UV-Vis spectrophotometer at a 480 nm wavelength.

### 2.7. In Vitro Cellular Uptake Behavior of DOX-MPN-HANP

To observe the DOX-MPN-HANPs’ internalization behavior towards cancer cells, we conducted an in vitro cellular uptake experiment using a squamous cell carcinoma (SCC7) cell line. In 6-well plates, 1 × 10^5^ SCC7 cells were seeded in each well and incubated in a humidified CO_2_ chamber for 24 h. The cells were then treated with a fresh RPMI medium (pH 7.4 or 6.5) containing DOX-HANPs or DOX-MPN-HANPs (10 µg of DOX per each group), followed by a 3 h incubation at 37 °C. Thereafter, the medium was removed, and the cells were washed twice using DPBS with Ca^2+^ and Mg^2+^. The cancer cells were fixed using a 4% paraformaldehyde solution, and the nuclei were stained using 4’,6-diamidino-2-phenylindole (DAPI). The cellular uptake behavior was observed using confocal laser scanning microscopy (CLSM, LSM 510 META NLO, Carl Zeiss GmbH, Jena, Germany).

To investigate the pH-sensitive internalization of the nanoparticles, we prepared fluorescein-labeled HANPs. In brief, 10 mg of HACA was dissolved in a phosphate buffer (pH 6.8), to which 20.21 mg of EDC and 12.13 mg of NHS were added and stirred for 30 min. Then, 1 mL of DMSO containing 9.15 mg of fluoresceinamine was slowly added and stirred overnight in dark conditions. The resulting solution was dialyzed against methanol and DW for two days using the membrane tube (molecular weight cut off = 12–14 kDa), followed by lyophilization to obtain fluorescein-labeled HANPs.

To further analyze the cellular uptake in a quantitative manner, we conducted a flow cytometry analysis. Briefly, 3 × 10^5^ SCC7 cells were seeded and treated with fluorescein-labeled HANPs or MPN-HANPs. The cells were washed twice with fresh DPBS and suspended in DPBS containing 1% FBS for single-cell analysis using flow cytometry (Guava Easycyte, EMD Millipore, Billerica, MA, USA).

### 2.8. Cytotoxicity of DOX-MPN-HANPs

We performed the CCK-8 assay to assess the cytotoxicity of the MPN-HANPs and DOX-MPN-HANPs. Briefly, SCC7 cells were plated on 96-well plates (1 × 10^4^ cells per each well) and incubated for 24 h. The medium was changed to an FBS-free medium (pH 7.4 or 6.5), which contained various concentrations of the HANPs, MPN-HANPs, free DOX, or DOX-MPN-HANPs. The cells were then incubated for an additional 24 h. After removing the culture medium, the cytotoxicity was evaluated using the CCK-8 assay, according to the manufacturer’s instructions (Sigma Aldrich Co., Saint Louis, MO, USA).

### 2.9. Statistical Analysis

The data’s statistical significance was determined using a one-way analysis of variance (ANOVA). A *p*-value of less than 0.05 was considered statistically significant (indicated with an asterisk mark). All experiments were repeated three times (*n* = 3).

## 3. Results and Discussion

Stimuli-responsive nanomedicines have been designed to elicit tumor-specific functions for treatment and/or diagnosis. In this regard, MPN would be highly useful to make pH-responsive nanomedicines since it can be formed as the diffusion barrier on the nanoparticular surface by the simple fabrication process [25]. Herein, in an attempt to develop the carrier of hydrophobic anticancer drugs for targeted cancer therapy, we have prepared MPN-HANPs which can release the drugs by recognizing the mildly acidic TME.

### 3.1. Synthesis and Characterization of MPN-HANP

**Figure 2:**
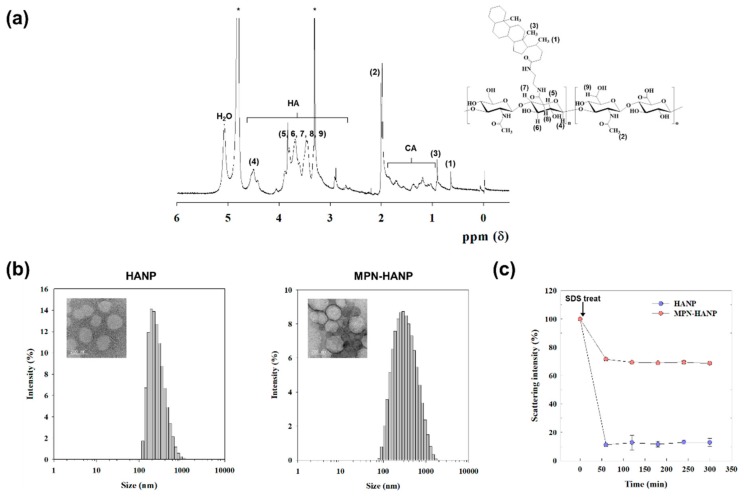
Physicochemical characteristics of the hyaluronic nanoparticles (HANPs) and MPN-HANPs. (**a**) ^1^H-NMR spectrum of HACA in *D*_2_O/C*D*_3_O*D* (1*v*/1*v*). (**b**) The representative particle size distribution of HANP and MPN-HANP. The insets are TEM images (scale bar = 200 nm). (**c**) Stability of HANPs and MPN-HANPs in SDS solution.

HA has several chemical functional groups including carboxylic acid, hydroxyl, and reducing end groups. Therefore, it is easy to chemically decorate it through various reactions. In this study, amine-functionalized CA was chemically grafted to carboxylic acids at the HA backbone (Figure 1). As shown in Figure 2, the chemical structure of the HACA was confirmed using a ^1^H-NMR spectrum, which shows a characteristic CA peak at 0.68 ppm (–C*H*_3_), and an HA peak at 2.0 ppm (–NHCOC*H*_3_). The degree of substitution of CA was estimated to 6.8, based on the integration ratio of characteristic peaks from CA and HA.

Owing to its amphiphilic nature, the HACA formed self-assembled nanoparticles and was readily coated by MPN in an aqueous condition. Both the HANPs and MPN-HANPs exhibited unimodal size distributions with a spherical shape (Figure 2b). The mean diameters of the HANPs and MPN-HANPs were 264.0 nm and 273.9 nm, respectively, indicating that there is no significant change in the size by surface modification (Table 1). The zeta potential value, however, significantly increased from −42.7 mV for the HANPs to −24.5 mV for the MPN-HANPs, implying that the surfaces of the HANPs were successfully coated by the MPN. In order to investigate the stability of the nanoparticles, the changes in the scattering intensity of the nanoparticles were evaluated in the presence of SDS as a function of time (Figure 2c). Upon addition of the SDS, a remarkable decrease in the scattering intensity (<15%) was observed for the HANPs, whereas a slight decrease (>70%) was found for the MPN-HANPs, suggesting the high stability of the MPN-HANPs.

### 3.2. In Vitro Drug Release Profile

**Figure 3:**
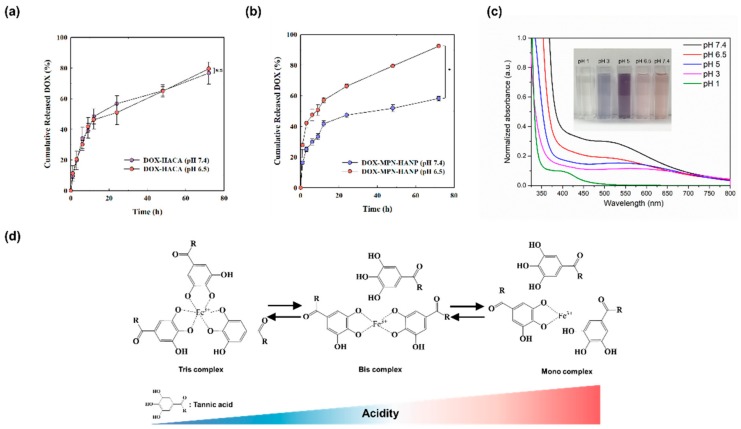
In vitro release profiles of doxorubicin (DOX) from (**a**) DOX-HANPs and (**b**) DOX-MPN-HANPs at different pH. (**c**) UV-Vis spectrum of the MPN layer. The insets are photographs of the MPN-dispersed solutions. (**d**) The coordination structures of MPN depending on acidity. Error bars represent standard deviation (*n* = 3).

To assess the tumor-specific drug release of the MPN-HANPs, we loaded DOX to the HANPs and coated it with MPN to form DOX-MPN-HANPs. The loading efficiencies, determined using a UV-Vis spectrophotometer, were 82.4% for the HANPs and 77.1% for the MPN-HANPs. This suggests that the loading efficiency is not significantly reduced by the surface-coating procedure because MPN is immediately formed on the nanoparticular surface after the addition of TA and FeCl_3_. We then investigated the drug release profile of DOX from the HANPs or MPN-HANPs in different pH conditions (Figure 3a,b). At both a pH of 7.4 and 6.5, the HANPs and MPN-HANPs rapidly released DOX for an initial 12 h, followed by slower release for the remaining period of time. Although the DOX-MPN-HANPs exhibited burst release of the drug, its amount was substantially lower than those reported elsewhere for polymer micelle-based formulations [34,35,36].

For the DOX-HANPs, the DOX release behavior was not significantly affected by pH. Interestingly, the DOX release from the DOX-MPN-HANPs was much faster in the mildly acidic solution (pH 6.5) mimicking TME, which might be due to the changes in coordination structure of the MPN [37]. This implies that the pH-sensitive MPN coating layer can play a role as the diffusion barrier of DOX, allowing for its tumor-specific drug release. To investigate the pH-sensitive characteristics of the MPN layer, its UV-Vis absorption behavior was assessed in different pH conditions since ligand-to-metal charge transfer (LMCT) bands of MPN were observed at 500~550 nm [35,36]. As shown in Figure 3c, the absorption intensity of the LMCT band was reduced at a lower pH by the disassembly of MPN coordination, implying that the MPN structure is highly sensitive to pH [25]. MPN consists of tris-complexes at the physiological condition (pH>7), whereas its coordination structure is changed to less stable bis- or mono-complexes at the acidic condition, thus facilitating diffusion of DOX from the nanoparticles (Figure 3d).

### 3.3. In Vitro Cellular Uptake Behavior

**Figure 4:**
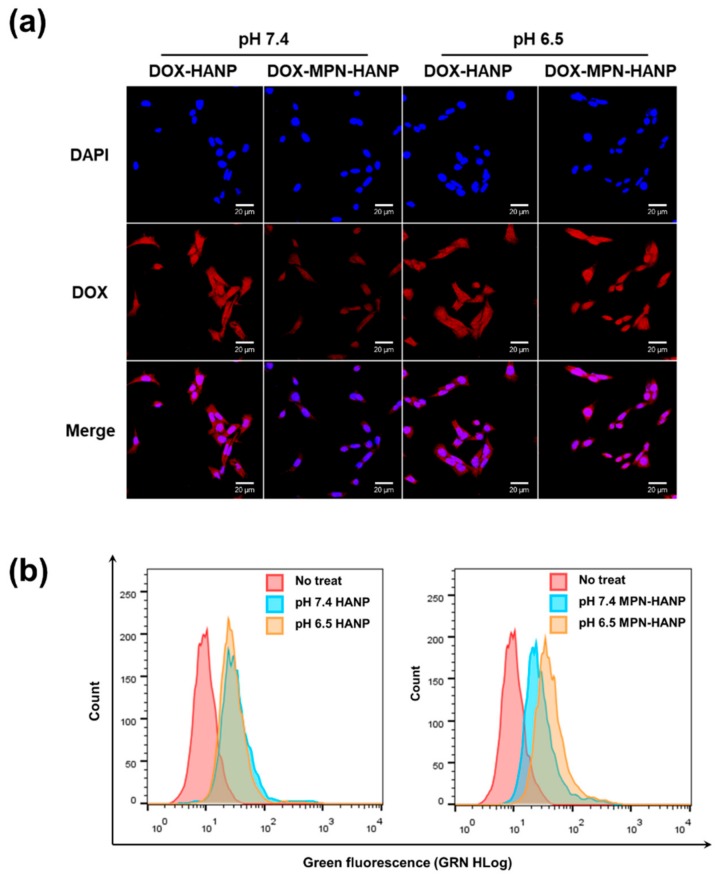
In vitro cellular uptake behavior. (**a**) Confocal microscopic images of HANP-DOX and MPN-HANP-DOX at pH 7.4 and 6.5 (scale bar = 20 μm). (**b**) Histogram of quantitative analysis by flow cytometry.

The cellular uptake of DOX was observed using confocal microscopy at different pH (Figure 4a). For the DOX-HANPs, strong DOX signals were observed at both pH 7.4 and 6.5, implying that DOX was effectively taken up by the cancer cells. Interestingly, the DOX-MPN-HANPs exhibited pH-dependent cellular uptake behavior of DOX. At pH 7.4, a weak DOX signal was detected at the intracellular level of SCC7 cells, whereas a considerable amount of DOX might be delivered at pH 6.5. The cellular uptake behaviors of the HANPs and MPN-HANPs were quantitatively analyzed using the flow cytometry (Figure 4b). For the HANPs, there were no significant differences in cellular uptake between pH 7.4 and. 6.5. On the other hand, compared to the physiological conditions at pH 7.4, the MPN-HANPs exhibited 1.72-fold higher cellular uptake at pH 6.5. These results suggest that, in the mildly acidic environment, the MPN-HANPs were taken up by receptor-mediated endocytosis, based on the specific binding of the HA surface to CD44 on the cancer cells. Since the MPN layer undergoes the structural transition from tris-complex to bis- or mono-complex under the acidic condition (Figure 3d), the HA surface might be easily exposed to facilitate receptor-mediated endocytosis.

For the facilitated delivery of DOX into the intracellular level, the nanoparticles should be effectively internalized. Since the DOX-MPN-HANPs have the pH-sensitive MPN surface layer which does not allow for receptor-mediated endocytosis at the physiological condition (pH 7.4), a stronger DOX signal might be observed at pH 6.5, in which the MPN layer on the HA surface is disassembled to facilitate the receptor-mediated endocytosis (Figure 3c). These results suggest that DOX-MPN-HANPs have the potential to facilitate receptor-medicated endocytosis for intracellular DOX release in the mildly acidic condition of TME.

### 3.4. In Vitro Cytotoxicity of DOX-MPN-HANP

**Figure 5:**
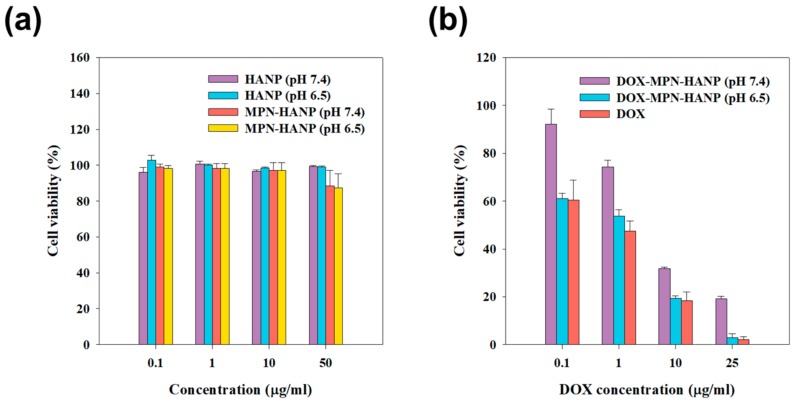
In vitro cytotoxicity effect. (**a**) HANP and MPN-HANP at pH 7.4 and 6.5 (**b**) MPN-HANP-DOX (pH 7.4 and 6.5) and free DOX (pH 7.4). Error bar means standard deviation (*n* = 3).

The in vitro cytotoxicity was evaluated using a CCK-8 assay by treating SCC7 cells with nanoparticles with and without DOX (Figure 5). Regardless of the pH, all the nanoparticles without DOX showed no significant cytotoxic effects over the whole range of sample concentrations, suggesting that the MPN chosen as the diffusion barrier was not toxic to the cells. As expected, the DOX-MPN-HANPs exhibited cytotoxic effects in a dose-dependent manner. Notably, the cytotoxicity was much higher in the mildly acidic conditions, allowing for the dissolution of the MPN layer as the diffusion barrier to facilitate the receptor-mediated endocytosis of the nanoparticles.

## 4. Conclusions

Introducing a tumor-specific function to nanoparticles is a critical step in developing a robust nanomedicine for cancer treatment. In this study, we explored the potential of MPN as an effective diffusion barrier on the HANPs for pH-responsive delivery of hydrophobic anticancer drugs. MPN-HANPs could selectively release DOX in the mildly acidic condition, mimicking tumor microenvironments. In contrast, a much slower release was found in the physiological condition (pH 7.4), implying that MPN-HANPs have the potential to deliver significant amounts of drug to the target site. Also, compared to the physiological condition (pH 7.4), the DOX-MPN-HANPs exhibited a higher cellular uptake of DOX at the mildly acidic condition (pH 6.5). For the clinical applications of DOX-MPN-HANPs, however, their particle size and burst release of DOX should be further optimized by reflecting the in vivo microenvironment of the human body. Overall, the results suggest that the MPN is highly useful to develop pH-sensitive nanoparticles in a simple and effective way for a tumor-specific anticancer drug delivery.

## Figures and Tables

**Table 1:** Characteristics of HANPs, MPN-HANPs, DOX-HANPs, and DOX-MPN-HANPs.

Sample	Hydrodynamic Size (nm) ^1^	Zeta Potential (mV) ^2^	DOX Feed Amount (%)	Loading Efficiency (%) ^3^	Loading Contents (%) ^3^
HANP	264.0 ± 13.11	−42.7 ± 0.38	-	-	-
MPN-HANP	273.9 ± 18.62	−24.5 ± 0.11	-	-	-
DOX-HANP	277.53 ± 5.20	−36.8 ± 0.47	10	82.4	8.24
DOX-MPN-HANP	271.3 ± 4.05	−28.07 ± 0.97	10	77.1	7.71

^1^ Average hydrodynamic size measured using dynamic light scattering. ^2^ Zeta potential was measured using a zetasizer. ^3^ Loading efficiency and loading efficacy were determined using a UV-Vis spectrophotometer. Error bars represent standard deviation (*n* = 3).
